# Shedding New Light on the 18th Dynasty Mummies of the Royal Architect Kha and His Spouse Merit

**DOI:** 10.1371/journal.pone.0131916

**Published:** 2015-07-22

**Authors:** Raffaella Bianucci, Michael E. Habicht, Stephen Buckley, Joann Fletcher, Roger Seiler, Lena M. Öhrström, Eleni Vassilika, Thomas Böni, Frank J. Rühli

**Affiliations:** 1 Department of Public Health and Paediatric Sciences, Legal Medicine Section, Laboratory of Physical Anthropology, University of Turin, Turin, Italy; 2 Centre for Ecological and Evolutionary Synthesis (CEES), Department Biosciences, University of Oslo, Oslo, Norway; 3 Laboratoire d’Anthropologie bio-culturelle, Droit, Etique & Santé (Adés), Faculté de Médecine de Marseille, Marseille, France; 4 Institute of Evolutionary Medicine, University of Zurich, Zurich, Switzerland; 5 BioArCh, University of York, York, United Kingdom; 6 Department of Radiology, University Hospital Zurich, Zurich, Switzerland; 7 Fondazione Museo delle Antichità Egizie di Torino, Turin, Italy; Hebrew University, ISRAEL

## Abstract

The mummies of Kha and his wife Merit were found intact in an undisturbed tomb in western Thebes near the ancient workers’ village of Deir el-Medina. Previous MDCT (this abbreviation needs spelling out) investigations showed that the bodies of Kha and Merit did not undergo classical royal 18th Dynasty artificial mummification, which included removal of the internal organs. It was, therefore, concluded that the retention of the viscera in the body, combined with an absence of canopic jars in the burial chamber, meant the couple underwent a short and shoddy funerary procedure, despite their relative wealth at death. Nevertheless, all internal organs - brain, ocular bulbs/ocular nerves, thoracic and abdominal organs - showed a very good state of preservation, which contradicts the previous interpretation above. In order to better understand the type of mummification used to embalm these bodies, both wrapped mummies were reinvestigated using new generation X-ray imaging and chemical microanalyses Here we provide evidence that both individuals underwent a relatively high quality of mummification, fundamentally contradicting previous understanding. Elucidated “recipes”, whose components had anti-bacterial and anti-insecticidal properties, were used to treat their bodies. The time and effort undoubtedly employed to embalm both Kha and Merit and the use of imported costly resins, notably Pistacia, do not support the previously held view that the two individuals were poorly mummified. Despite a lack of evisceration, the approach clearly allowed their in situ preservation as well as affording a fairly successful mummification.

## Introduction

On February 15 1906, the archaeologist Ernesto Schiaparelli (1856–1928) and the Inspector of Antiquities, Arthur Weigall (1880–1934), opened the door of the inner chamber of an unplundered tomb (TT8) located on the cliffs surrounding the ancient village of Deir el Medina, Egypt. Tomb TT8 was revealed to be the burial and undisturbed resting place of the 18th Dynasty royal architect Kha and his wife Merit, the Mistress of the House [1].

The name of the royal architect, his social status, and details of his private life, were already known to scholars by the early years of the 19th century, Bernardino Drovetti (1776–1852) having discovered Kha and Merit’s decorated funerary chapel, surmounted by a pyramidion, and their funerary stele on the top of Deir el Medina’s western cemetery. Kha and Merit’s stele (Turin Museo Egizio Cat. 1618 RCGE 5673) was already part of the Drovetti Collection at the Turin’s Egyptian Museum circa 80 years before the tomb itself was uncovered [1–3].

Due to its unexpected location, some 25 meters north of the family funerary chapel (coordinates apr. 25.7°N 32.6°E), the tomb of Kha and Merit, the most intact non-royal tomb from the New Kingdom, had never been violated. Along with the two large wooden sarcophagi containing the mummies of the architect and his wife, over 500 hundred items, all of which would have served Kha and Merit in the Afterlife, were recovered from the tomb. As one of few Egyptian burials to remain entirely undisturbed since the time it was sealed, the discovery is of greatest importance for the reconstruction of funerary customs of the New Kingdom. It also represents a seminal contribution to a knowledge and understanding of funerary customs of non-royal individuals at this time. [4]

Despite ‘Egyptian mummification’ often being seen as a very narrow and specific definition, even within the study of ancient Egypt itself, the reality is that it evolved, and indeed regressed, over time. Within this there were inevitable variations, despite some general consistencies, and as a process it was confined to the privileged elite for much of its history—through whose eyes much of the received wisdom continues to be seen—with the particular status of individuals inevitably affecting the treatment they ultimately received.

When considering the available non-scientific evidence, there are few ancient Egyptian texts on embalming, all of which are late in date, when Egypt was no longer governed by a native monarchy. Outside these sources, secondary textual evidence for mummification is provided by the classical writers Herodotus [5], Diodorus Siculus [6], Strabo [7] and Pliny [8], although these are also relatively late in date. The most important of these is that of Herodotus [5] (c. 450 BC), although archaeological evidence shows that even this earliest of accounts was centuries after the high point of the embalmers’ ‘art’ c.1350 BC [9,10]. Consequently, the lack of a proper and full written record of the process by the ancient Egyptians themselves means the nature of the embalming agents in particular, remains obscure.

Where scholarly and scientific studies on Egyptian mummies have been carried out they have historically tended to have an anatomical and medical perspective [11,12], which while unquestionably providing valuable insights has necessarily limited the questions it is possible to answer, particularly in connection with the embalming materials themselves. Again, although providing valuable information on funerary practices and disease, studies of Egyptian mummies largely continue to exclusively employ imaging techniques; X-raying, CT scanning and MRI [13–16], which necessarily cannot identity the embalming agents themselves.

Although relatively modest in number, modern investigative chemical techniques applied to securely provenanced and dated mummies and their embalming materials [17–22] have provided insights into the complex organic materials used in mummification during Egypt’s pharaonic period (c. 2900–332 BC) [23]. The relevance of these chemical studies for Egyptian mummification and embalming has also been further demonstrated recently with the investigation of prehistoric Egyptian burials pushing back the origins of Egyptian mummification by some 1500 years to the Late Neolithic period [24].

On New Kingdom mummification specifically, it is seen as a time when embalming became relatively standardised [25], involving, in order: removal of the brain, removal of the internal organs, sterilization of the body cavities and internal organs, embalming the internal organs, temporarily packing the thoracic and abdominal cavities, dehydrating the body with the salt natron, removing the temporary packing, repacking the body with permanent packing material, anointing the body, coating the body with embalming ‘resin’, and wrapping the body in linen, with amulets and embalming agents applied to the linen as the body is wrapped. Although the omission of some or all of these processes for a ‘traditional’ Egyptian mummy has been considered previously [26], a focus on royal mummies in particular means there is less of an understanding of the exact procedures and materials applied to individuals of the lower elite during this time, and where their study has occurred, a rigorous investigation of the embalming materials employed has not been part of the research [26]. This has led to the erroneous assumption that the lower Egyptian elite were not embalmed, which given the symbolic importance of the process is of fundamental significance.

Here we employ an investigative approach combining the benefits of imaging techniques with those of chemical investigations in order to provide truly meaningful insights into not only some of the physical procedures employed by the embalmers, but also—and crucially—the nature and identity of the embalming agents themselves.

If the embalming procedures used to preserve Kha’s and Merit’s bodies are compared with those used for Yuya and Thuyu, Queen Tiye’s parents, sound differences emerge. Yuya and Thuyu’s tomb (QV36) represent one of the few non-royal burials from the Valley of the Kings dating back to the late 18th Dynasty. Although their tomb was plundered several times in antiquity, the mummies have not substantially been damaged.

Recent MDCT investigations carried out on the Royal Mummies (18th to the 20th Dynasties) showed that both Yuya and Thuya underwent excerebration and evisceration although to a different extent. Sagittal CT reconstruction in midline of head of Thuya (18th Dynasty) showed a defective base of anterior cranial fossa. The skull showed to be almost empty with a complete removal of brain except from a few dural remains. No embalming materials were introduced intracranially. An attempt to remove Yuya’s brain was performed, similarly, by using the transnasal route. However, only a small portion of the brain was extracted, the majority of the desiccated brain having been left in situ and treated with resinous compounds [27]

Similarly, Saleem and Hawss have provided evidence that Yuya and Thuyu had been eviscerated and differently packed so to provide them with a life-like fullness of their faces and bodies, The use of subcutaneous packing in Yuya and Thuyu’s mummies had already been described by G. E. Smith who could, however, only perform a visual inspection. CT scanning allowed to see the different distribution of the subcutaeous packing. While Yuya’s remains were extensily packed (face, neck and torso), the use of subcutaneous packing in Thuyu was limited only to her face [28].

Previous investigation indicated that neither Kha nor Merit had undergone the classical 18th Dynasty artificial mummification seen in royal mummies of the period. The findings presented produced a series of speculations including the suggestion that both corpses, although adorned with beautiful jewellery, had undergone a shoddy funerary treatment [29]. Indeed, it has been accepted that Kha and Merit underwent no embalming or mummification at all, but were merely wrapped in linen [30,31].

The aim of the reassessment was to provide new insights into the thanatological treatment of these non-royal elite, individuals, which resulted in the moderately successful preservation of their corpses some c. 3400 years later.

### The historic background

Kha is thought to have originally been a man of modest background who may later have enjoyed a successful career on the basis of merit. He rose to the rank of Director of the Royal Works in Deir el Medina where he oversaw the construction of the royal tombs. He served three 18^th^ Dynasty Kings: Amenhotep II (1424–1398 BC), Thutmose IV (1398–1388 BC) and Amenhotep III (1388–1348 BC) and died during the reign of the latter Pharaoh. From the style of the funerary objects found in the burial chamber, Kha’s death occurred a number of years before those of Yuya and Thuju—Queen Tiyi’s parents, the spouse of King Amenhotep III—whose plundered tomb (KV46) was discovered by Quibell in the Valley of the Kings on February 5 1905 [32].

**Fig 1:**
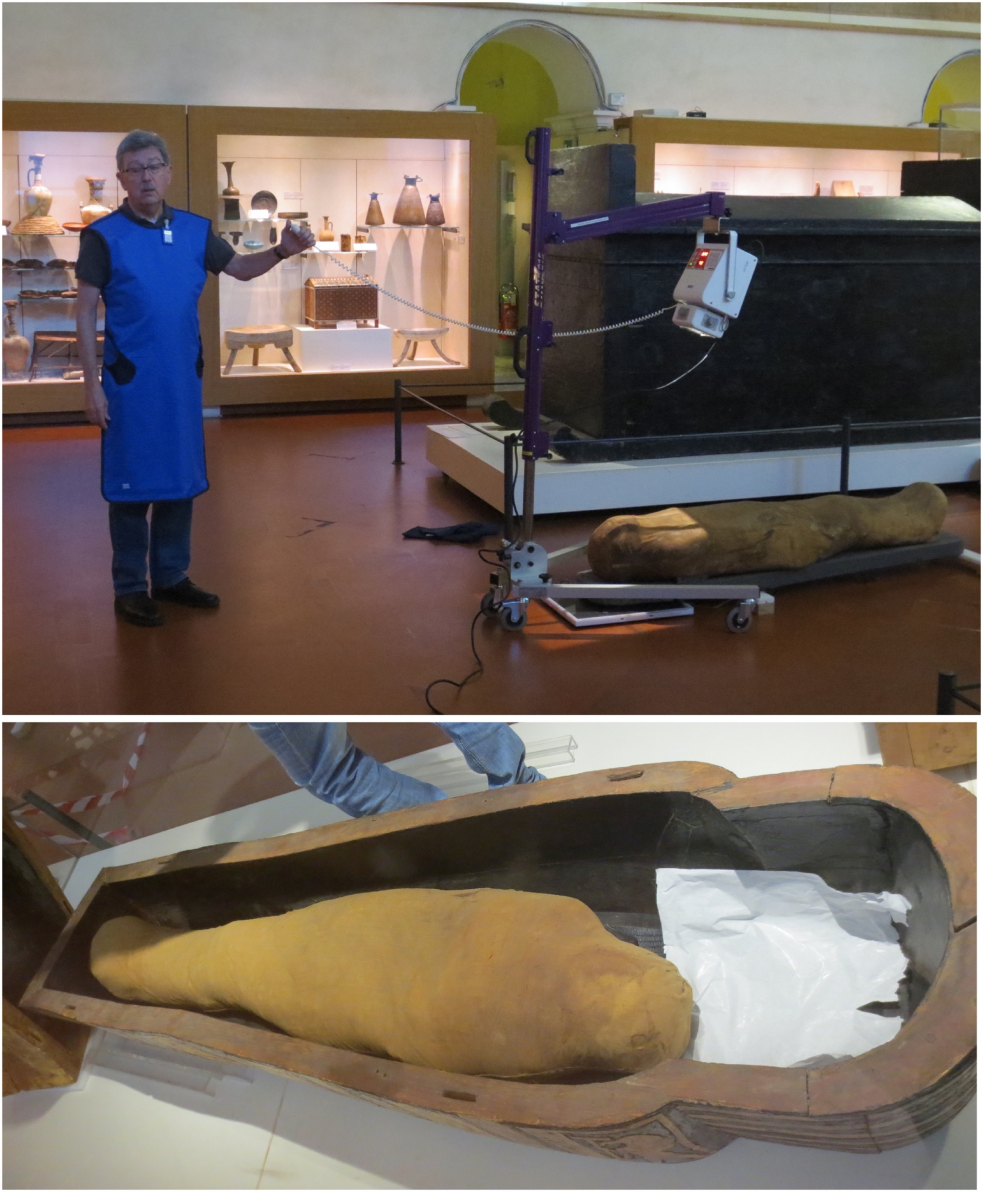
Digital X-raying of Kha (upper); the mummy of Merit (lower). Merit’s coffin, which is too large for her size and originally belonged to her husband.

Based on the items found in their burial—500 objects including five nested coffins, some of which were gilded, full sets of linen clothing and monogrammed underwear, a variety of types of food and two of the earliest known examples of the Book of the Dead, complete with vignettes, it can be assumed that Kha and Merit were a wealthy couple. They had three known children: two sons, Amenemopet, Nakhteftaneb and a daughter, Merit II. Amenemopet followed his father’s career while nothing is known about Nakhteftaneb. Merit II became a Singer of Amun. All of them survived their mother [33]. Merit passed into the realm of Osiris, Lord of the Dead, long before Kha. Her own coffin was probably not finished when she died unexpectedly, since she was buried in Kha’s anthropomorphic coffin. Due to her being much shorter than her husband, Kha also donated some of his monogrammed linen fabric so that the corpse could be safely accommodated inside the coffin (Fig 1). Merit’s inner coffin was then placed into a black shiny rectangular outer coffin. The two anthropoid coffins of Kha are of exceptional quality. His outer coffin is also covered with a shiny black coating, while the face, the hands, bands of inscriptions and figures of funerary deities are of gilded gesso. The inner coffin is fully gilded. These coffins are extremely similar to those of Yuya and Thuyu [32]. The shiny black material covering the outer coffins of both Kha and Merit has previously been described as ‘bitumen’ [1,3], but this has been revised as part of this study.

## Materials and Methods

The catalogue numbers of the investigated mummies are:

1. Mummy Turin Fondazione Museo delle Antichità Egizie N 13015 Suppl. 8431 from tomb TT 8, identified as Kha;
2. Mummy Turin Fondazione Museo delle Antichità Egizie N 13016 Suppl. 8471 from tomb TT 8, identified as Merit.

Over 500 items found in Kha’s and Merit’s burial chamber, which bear their names, and that are currently exposed in their reconstructed tomb in Turin’s Fondazione Museo delle Antichità Egizie were observed in order to gain further information on their social status and in life possessions [1]

Since the corpses of Kha and Merit are completely wrapped, their investigation relied upon the use of non-invasive imaging technology, minimally destructive analysis of small areas where some limited damage had already occurred, and small samples from Kha and Merit taken historically since their arrival in Turin in 1906 and held in museum storage. This approached aimed to maximise information gained while minimising the impact on the invaluable mummies of Kha and Merit. Both mummies had been previously subjected to conventional X-rays in 1966 [34,35] and Multidectector Computed Tomography (MDTC) in 2001 [29,36]. The integral 2001 CT scan dataset of images is not publicly accessible. Therefore, direct comparisons between the CT scan images and current X-ray imaging couldn’t be performed. The previous CT report suggested that both corpses underwent a shorter thanatological treatment, presumably compared to royal individuals of the period, but still allowed all inner organs—brain included—to have been preserved reasonably well [29]. Nevertheless, there was no meaningful attempt to fully explain the nature and significance of this ‘shorter procedure’ with respect to the possible practical and religious significance of this unusual type of mummification.

In January 2014, a state-of-the-art mobile digital X-ray imaging (EXAMION PX 60 HF; max. output 3.2 kW, Voltage range 40–100 kV, Exposure range 0.4–100 mAs) was performed in situ at Turin’s Fondazione Museo delle Antichità Egizie (Fig 1).

The new set of non-invasive investigations detailed above was coupled with minimally invasive organic and inorganic chemical investigations. These were performed on very small samples (typically ~0.1 g) from both individuals outer wrappings, and material in museum storage, shedding significant new light on their mummification process. Very small samples of the shiny black coating covering the outer coffins of both Kha and Merit were taken from each to establish the nature and origin of the material and to determine whether both coatings of ‘black bitumen’ were related in composition and technological production.

## Results

### The mummy of Kha (Turin N 13015 Suppl. 8431)

#### Previous investigations (1966, 2001)

Kha was laid out in a supine position with his arms extended along the sides and the hands laid flat over the pubis [29]. Previous stature estimates indicate, respectively, that the architect was approximately 171 cm [29] to 172 cm tall [34]. Martina et al. described Kha as a possibly overweight and diseased man of circa 60 years of age at the time of his death, affected by diffuse atherosclerosis and cholecystitis [29,36]. The state of skeletal preservation was described as excellent whereas the dentition was described as generally poor. An intra-vitam fracture of the L1 vertebra and severe arthritis level with spine and knees were also described [29].

With regards to the funerary treatment, previous reports indicate that Kha had neither been excerebrated nor eviscerated. Due to this and to the absence of canopic chest associated with the burial, it was claimed that his corpse underwent a “poor standard of mummification”. Although it was claimed that his corpse was poorly mummified, the preservation of the internal organs—brain included—was reputed to be optimal [29].

### Re-assessment of Kha’s mummy (2014)

#### Stature, age and pathologies

**Fig 2:**
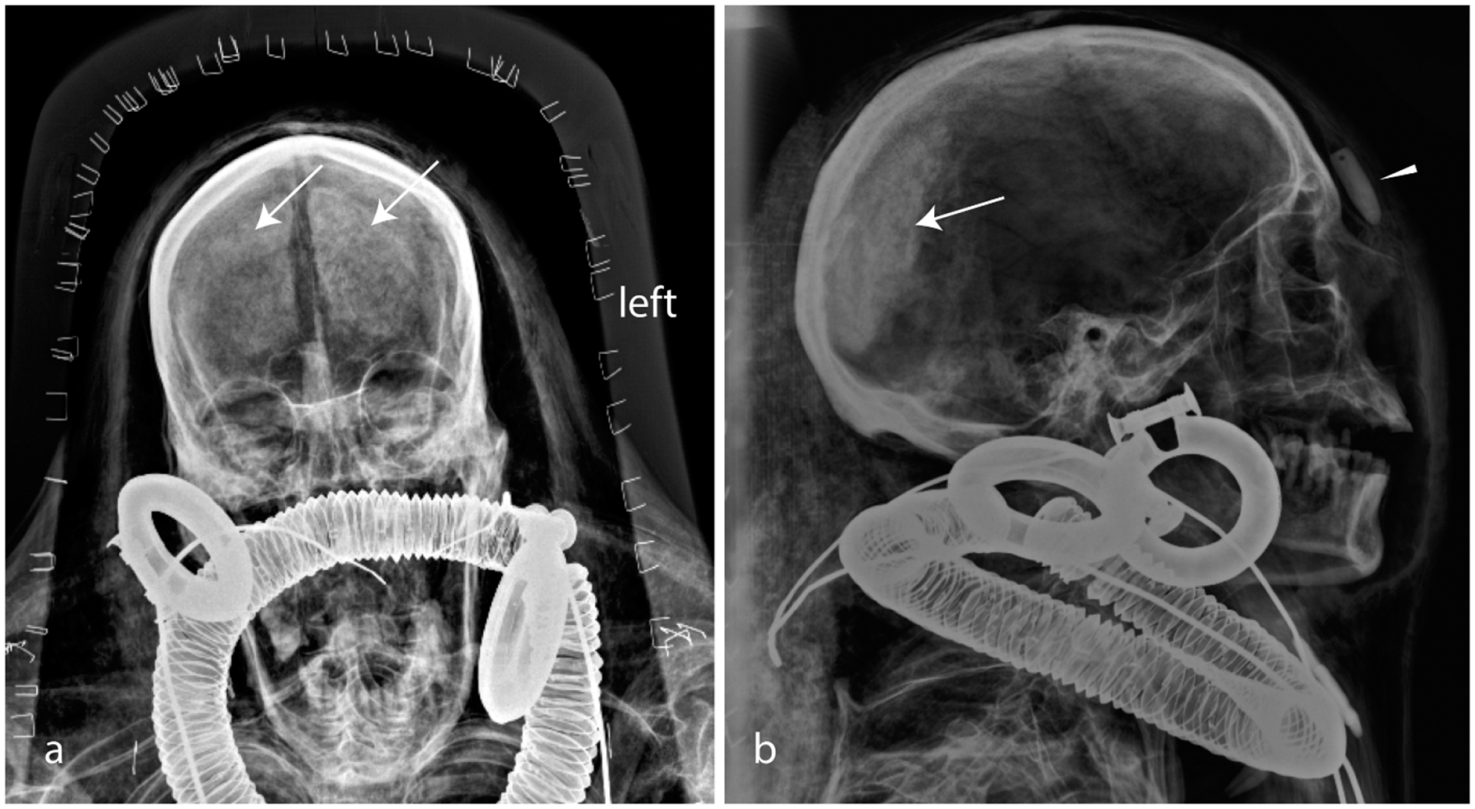
Kha’s skull a) frontal (ap) and lateral b). Note the shrunken brain remnants (arrows). The shape of the nasal bone indicates an aquiline profile. A snake’s head (made of stone) is clearly visible at Kha (arrowhead, lateral view). A dense oval plate (amulet?) is visible above the “Gold of Honour” collar. The collar of honour is made of gold discs. The broad earrings are made of ca. 1 mm thick gold foil. Imaging parameters a): 70kV, 3,2mAs; b) 70kV, 8mAs.

**Fig 3:**
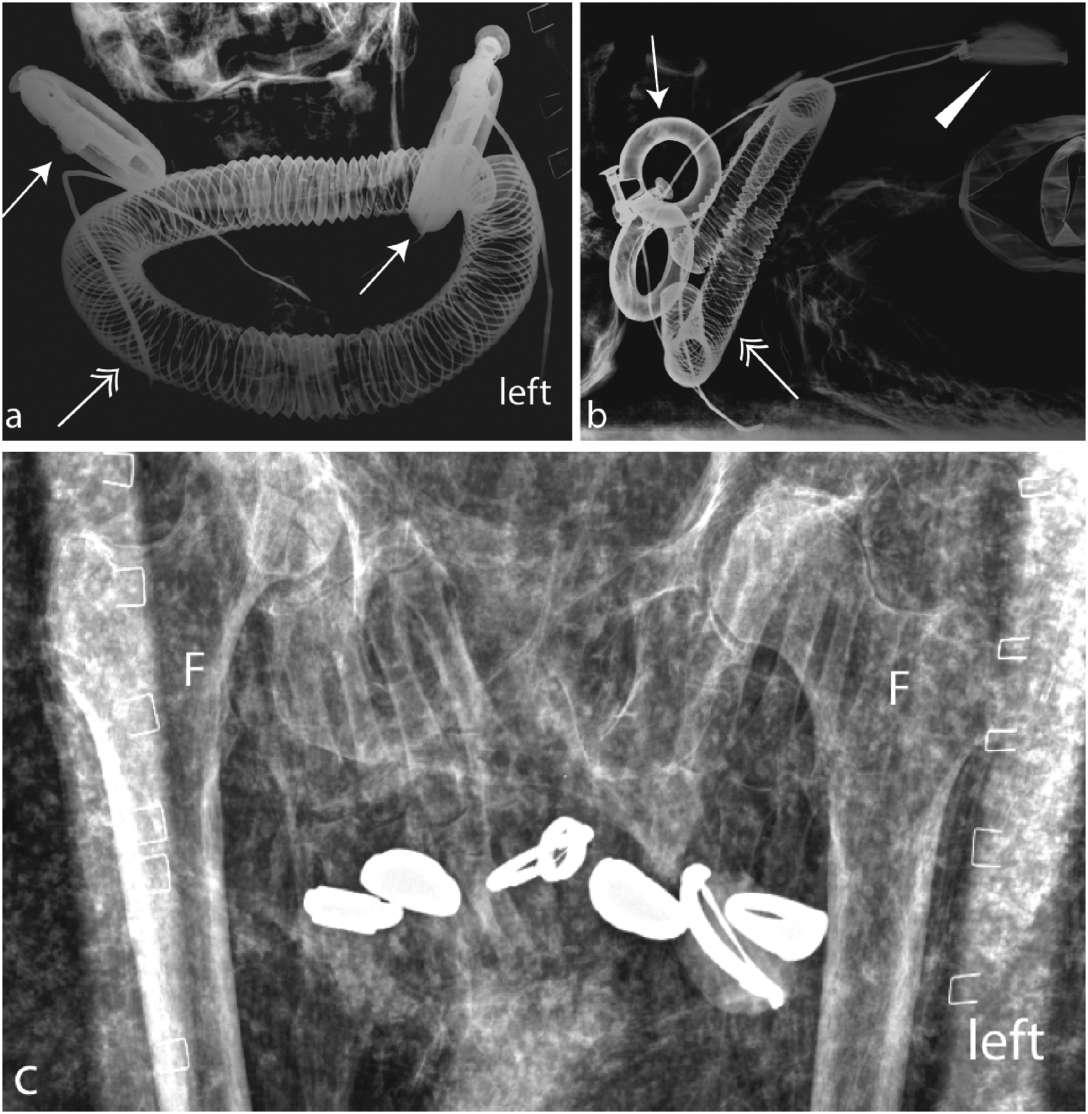
Kha’s jewellery: a, b) Kha wears a wide collar (double arrow), large ear-rings (arrows) and a large heart scarab on a metal rod (arrowhead); c) the hands laid on the pelvis. Six finger rings are visible. F. Femoral bone. Imaging parameters: a): 80kv, 8mAs; b): 100kV, 10mAs; c): 70kV, 5mAs.

The mummy of Kha measures 24 x 43 x 168 cm (depth, width, length). He was a mature adult in his fifties to sixties when he died (Figs 2 and 3) based on the extensive alterations of the lumbar spine and the poor dental health. His suggested biological age at death is consistent with the known biography.

**Fig 4:**
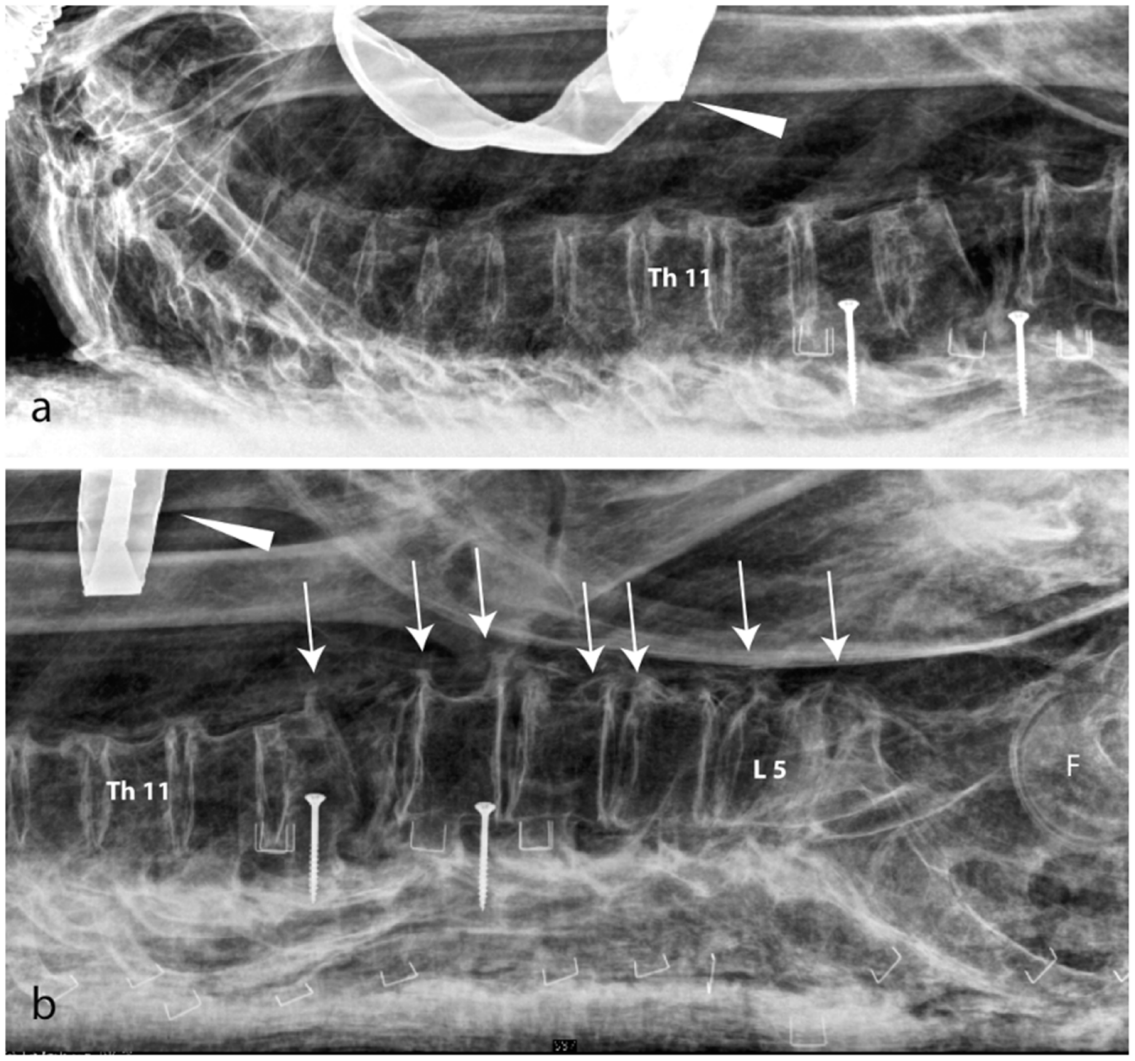
Kha. Lateral view of the thoracic (a) and lumbar (b) spine. The vertebral column shows a dislocation between L1 and L2 (post-mortal change). Degenerative changes (spondylophythes, arrows) are visible in the lower lumbar vertebra, suggesting that Kha was of advanced age. Arrowhead: funerary jewellery, as seen in Fig 3. F: femoral head. Imaging parameters a): 70kV, 8mAs, b): 100kV, 8mAs.

**Fig 5:**
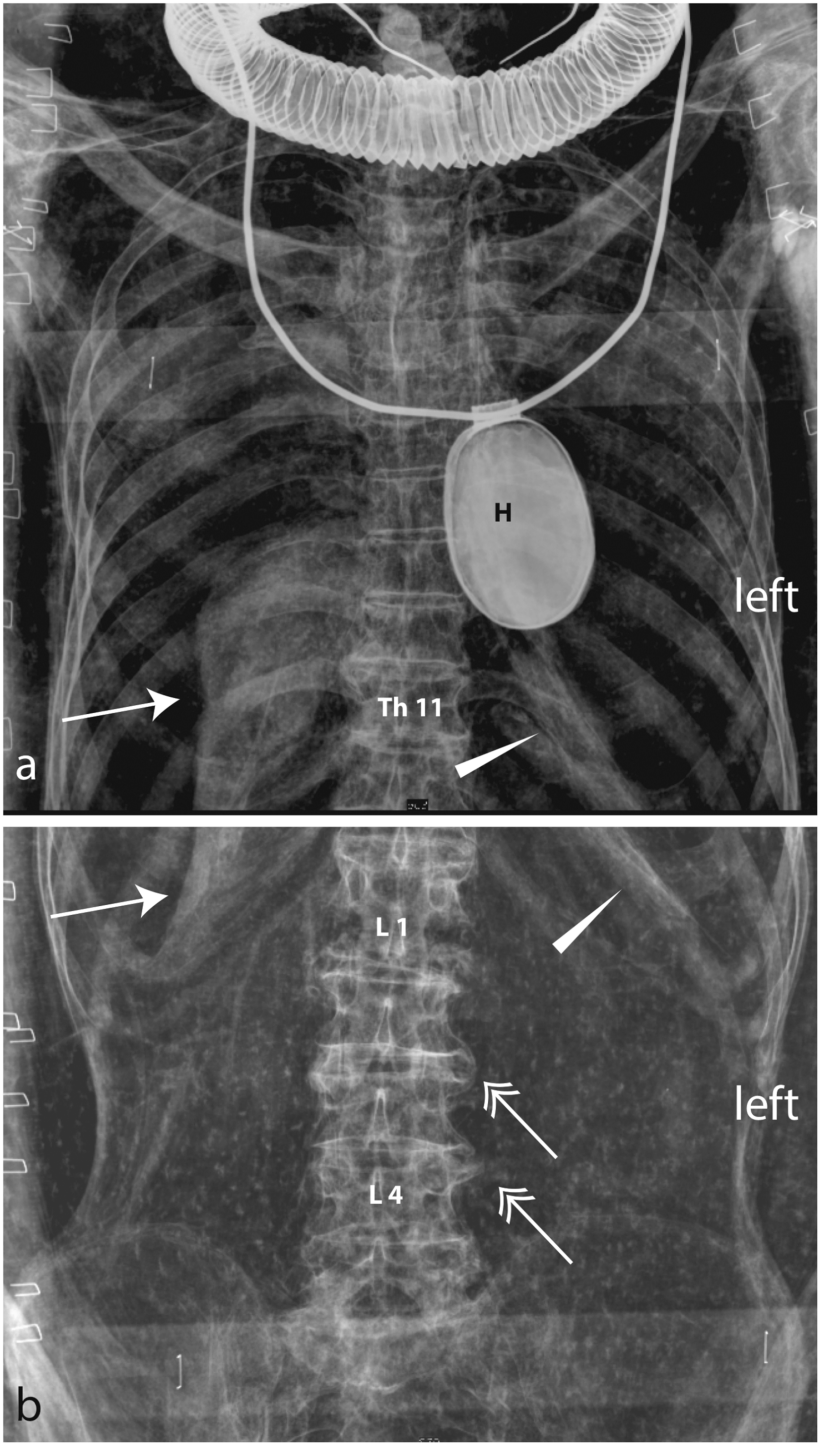
Kha. Frontal view (ap) of the thorax/abdomen. Possible remains of the shrunken lungs (arrow) and a possible bronchus rest (arrow head) are observable. The lower lumbar vertebrae show degenerative signs (spondilophythes on their left side). H: heart scarab. Imaging parameters: a & b): 80kV, 8mAs.

Except for a few pathological conditions observed on the spine (Figs 4 and 5), Kha’s skeleton can be compared to that of a modern day healthy old man. An unusual structure is observable level with the ventral parasternal side of the first rib (Fig 5); this can be either due to a bone overgrowth or to the effect of overlaying soft tissue. Due to the unavailability of previous CT scan images, more in-depth speculations to ascertain the nature of the above structure cannot be performed. Possible remnants of the bronchi and shrunken lungs can also be seen on the X-rays (Fig 5).

**Fig 6:**
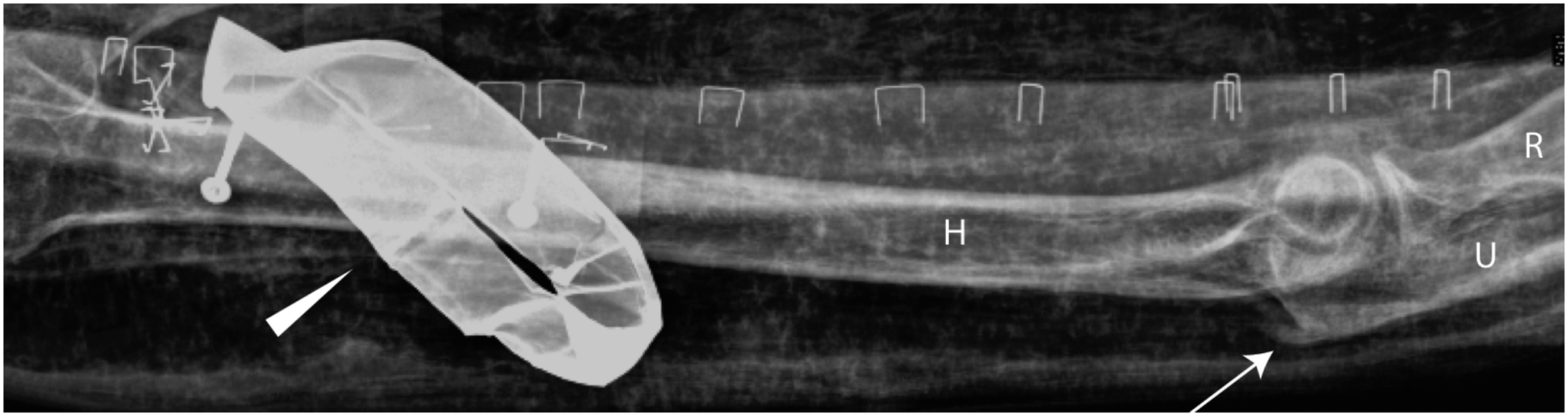
Kha: The right elbow shows an obvious enthesopathy of the insertion of triceps brachii muscle (arrow). Arrowhead: A golden foil was wrapped around his upper arm (funerary jewellery). F: humeral bone. U: ulnar bone. R: radial bone. Imaging parameters: 70kV, 3,2mAs.

At the right elbow, an enthesopathy is evident at the insertion of the triceps brachii (Fig 6); signs of spondylosis with several osteophytes are observable over the entire lumbar spine (L1/2, L2/3, L3/4, L5/S1) (Fig 5).

The L1 vertebra (first lumbar) is malpositioned and deformed with the distal end plate slightly oriented towards the left. A gap in the L1 vertebra, which also affects the Th12 (the last thoracic vertebra), is observable and is linked to post-mortem damage (Figs 4 and 5). Some slight degeneration is visible in the knees. The cause of Kha’s death remains unknown, there is no evidence of a fatal trauma.

#### Kha’s dentition

Anterior-posterior, lateral and half-axial projections, although not ideally suited for diagnosis of dental pathologies, were performed in order to gain overall information on the state of preservation of Kha’s dentition (Fig 2). The mouth of Kha is open to a significant extent. In the upper jaw, the central upper frontal incisors according FDI classification [37] (11; 21) and lateral (12; 22) incisors are still preserved although both lateral incisors (12, 22) seem to have peri-apical osteolysis. Upper premolars and molars, except some root remnants, were lost intra-vitam. No post-mortem tooth loss could be identified within the bandages. Because of the missing teeth in the upper maxilla, the dentition was severely limited in its functions.

In the lower right mandible, teeth are present from the incisors to the first premolar (41,42, 43, 44, 45) and on the left side to the elongated second molar (lower left side 31,32,33,34,35,37 and upper left 26). In these X-rays, carious lesions were not detected and the status of the periodontium could not be evaluated. Because of the missing teeth in both the upper and lower jaws, the dentition was severely limited in its functions. Thus, an overall poor state of preservation of Kha’s dentition was observed.

### The mummy of Merit (Turin N 13016 Suppl. 8471)

#### Previous investigations (1966, 2001)

Merit was laid out in supine position with extended arms and the hands nearly crossed over the pubis [29]. Previous stature estimates indicate, respectively, that she was 148 cm [29] or ca. 160 cm tall [34]. Based on the relative complete dentition, Martina et al. estimated that Merit died aged 25–30 years old. CT scans showed that, similarly to Kha, she had been neither excerebrated nor eviscerated. Eye bulbs could not be identified as artificial eyes, made of a radio-opaque material, were positioned on the orbits before the funerary mask was positioned on her face and chest. The state of preservation of the skeleton was judged as poor; this was due to a series of possible post-mortem fractures and resulting displacement of the bones, especially those level with the chest and the pelvis.

### Re-assessment of Merit’s mummy (2014)

#### Stature, age and pathologies

**Fig 7:**
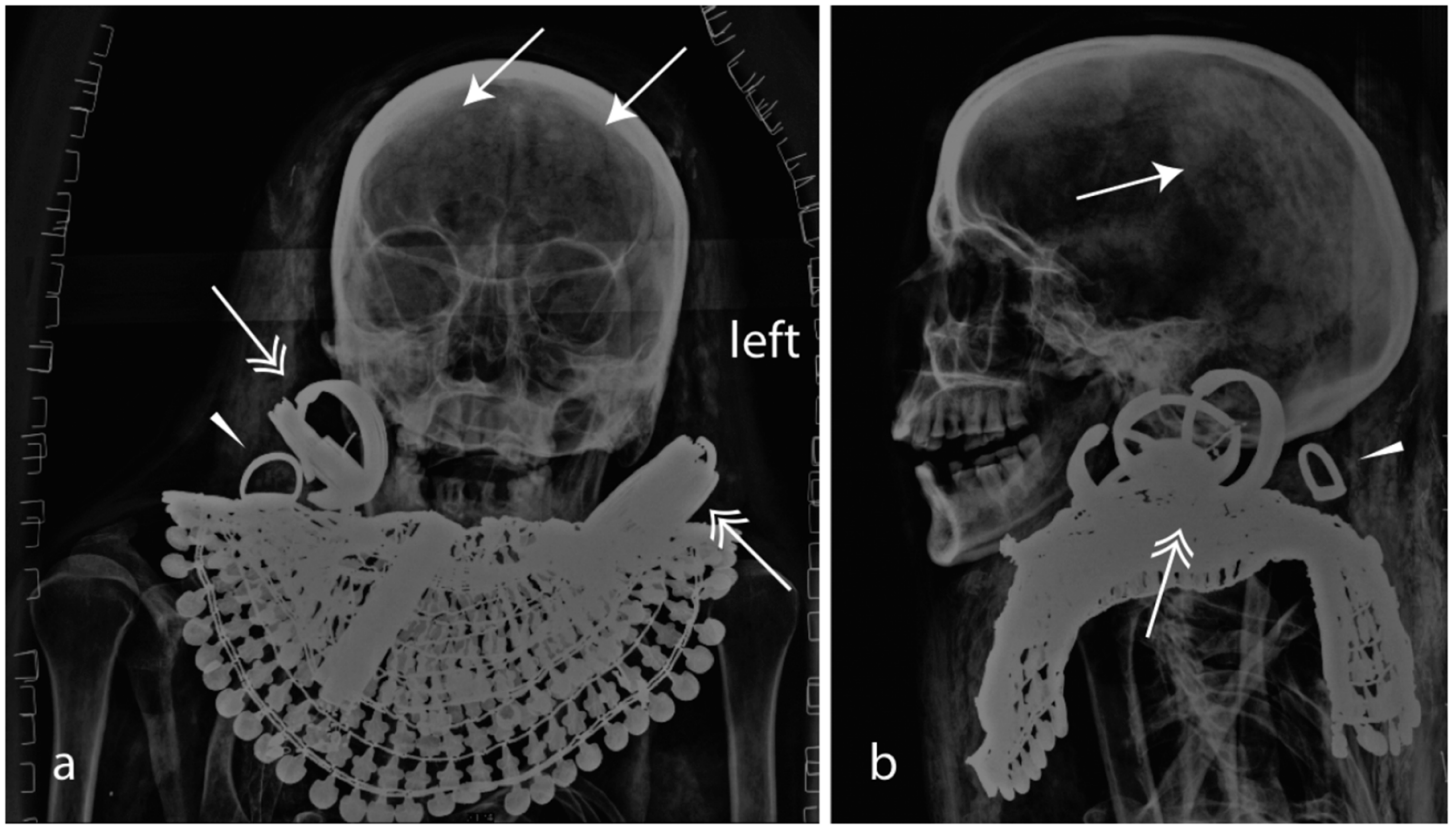
Merit: a) frontal view and b) lateral view of the head. Inside the cranium, remnants of the shrunken mummified brain are visible (arrows). The dentition supports the suggested age of ca 30 years. Merit probably also had a prominent aquiline nose. An Usekh-collar, two ribbed earrings (double arrow) and a dislocated finger ring that moved behind the neck (arrowhead) are observable.

The remains of Merit measure 10 x 34 x 147 cm (depth, width, length). She was an adult when she died and, based on the absence of degeneration of her bones, her age at death can be placed at approximately 25–35 years old (Fig 7).

The tibia of Merit measures on X-ray c. 42.5 cm. Using the X-ray correction factor (c. 1:1.15), the true length of the tibia is approximately 37 cm. Using different body height reconstruction formulae, the realistic body height of Merit is estimated to between 149 cm [29,34,38] (Trotter Gleser, female Caucasian) and c. 162 cm [39] (Pearson female, prehistoric). The female Black African formula of Trotter and Gleser producing an unrealistic tall reconstruction of c.168 cm by over-emphasizing the distal limbs. Therefore a stature of 160 cm appears to the more realistic [40].

**Fig 8:**
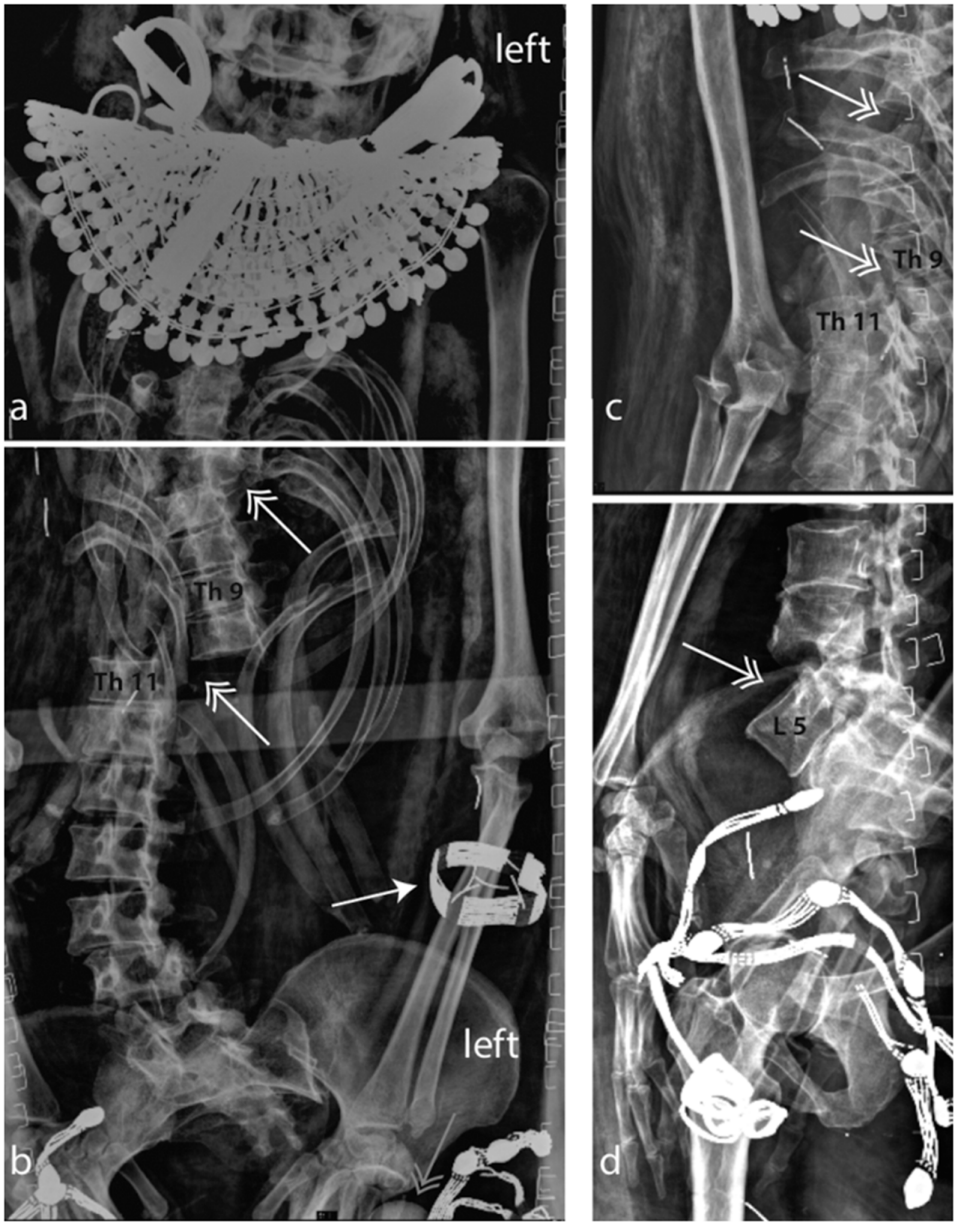
Merit. a) frontal and b) lateral view of the vertebral column and the pelvis. A massive post-mortal disruption of the vertebrae (double arrows) and ribs are visible. The left hip is luxated (red double arrow). No degenerative were observed. Merit wears a fine bracelet on her lower arm (arrow).

**Fig 9:**
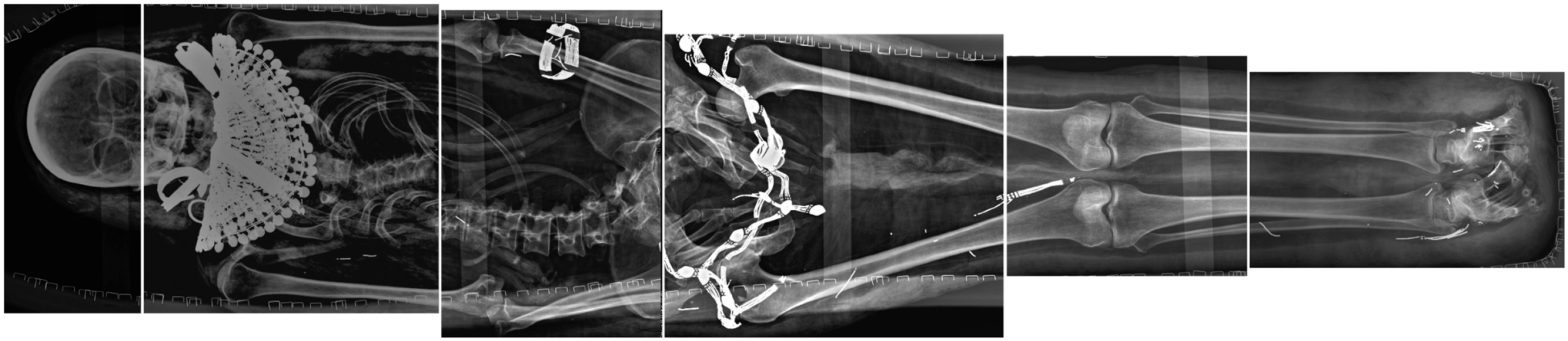
Merit’s total skeleton reconstruction.

The mummy shows very significant post-mortem damage. The thorax is massively depressed and the rib cage is broken (fallen ribs can be identified in the abdomen) (Figs 8 and 9). At the right elbow, a luxation of the radius and ulna is observable and the right wrist is luxated. The spine is disrupted level with T10/1 and L4/5 and the vertebrae T11/L3 are dislocated and rotated axially apr. 45°. The sacro-iliac joint shows a dislocation and the pelvis is disrupted (luxation of the left hip and disarticulation of the sacrum) (Figs 8 and 9). Merit’s cause of death is unknown. The unusual dislocation of the ribs and of the vertebral column argues against an intra-vitam trauma.

#### Merit’s dentition

Anterior-posterior, lateral and half-axial projections, although not ideally suited for diagnosis of dental pathologies, were again performed in order to gain overall information on the state of preservation of Merit’s dentition. Merit’s mouth is slightly open. In the maxilla, a premolar (15) and the last molar (18) were lost ante-mortem; upper left premolar (25) shows a deep carious lesion on its mesial and distal surfaces and the second molar (27) possibly on its distal surface. In the right mandible the lower right canine teeth (43), and the subsequent premolars (44 and 45), and on the left lower side the first premolar (34) were probably lost intra-vitam. According to Melcher et al. [41], the abrasion of the visible wisdom teeth (38, 48 and 28) is grade 1 whereas the other molars display grade 2–3. Only minor alveolar bone loss is observable. Apart from some impairment, her dentition was still functional.

### The jewellery of Kha and Merit

In order to acquire further details on the construction of the jewellery, the X-ray analyses were focused on using energies (80-100kV instead 60kV) most suitable for the materials they were likely to be more dense (e.g. made of metals). The type of metal commonly used in high rank burials was gold. This statement is fully corroborated by textual evidence and by comparisons with similar jewellery found in royal tombs dating to the same period (e.g. Amenhotep III). Kha and Merit died during Amenhotep III’s reign and were highly reputed members of their community. Therefore, since they were 18th Dynasty high status individuals, it can be confidently assumed that the jewels they wear are made of gold as well [42].

#### Kha’s jewellery

Kha wears extensive and large-sized jewellery (Figs 3 and 5) and he was probably dressed in a kilt or a bag-tunic following the New Kingdom’s fashion [43–45]. A detailed list of his ornaments is given below.

#### Golden ear-rings (jewellery worn in life and death)

Kha is one of the earliest known examples of an Egyptian man wearing large ear-rings [35,42]. The two half-shaped earrings are made of gold sheet, 1mm thick (Fig 3). This design was very widespread during the 18th Dynasty [42,44,46,47] and the custom of wearing ear-rings, which appeared at the dawn of the New Kingdom and this fashion was likely introduced from Nubia [35]. Two examples of a similar type of ear-rings have been identified in a 17^th^ Dynasty female burial in Qurna [42] and in an 18th Dynasty burial [46].

#### Gold of honour collar (jewellery worn in life and death)

Kha has a ´Gold of Honour´ collar, the prestigious reward that distinguished ancient Egyptians received from their King. It is made of a single string of golden discs [33,48] (Fig 3). A five-string ´gold of honour´-collar was identified in King Psusennes I’s Tanis Tomb III (Cairo Mus. JE 85751) and weights 6.315 kg [49,50]. It is therefore be estimated that the weight of Kha’s collar ranges between 1–1.5 kg.

The ´gold of honour´-collar is widely represented in the iconography (e.g. statuary, reliefs, wall paintings), for example Sennefer’s three-row collar in a wall painting in his tomb TT 96 [51,52] or the four-row collar he wears on his statue dyad with his wife Senai (Cairo Mus. JE 36574 CG 42126 [51]. Men and women alike received this royal award as shown in the Amarna tomb reliefs of Ay and his wife Ty, who are both decorated with ´Gold of Honour´ collars given by King Akhenaten [48,53]. Horemheb received several of these awards when he was a general serving Tutankhamun as wall reliefs from his Saqqara tomb attest [48,54].

#### Six finger rings (jewellery worn in life and death)

Previous X-rays identified five finger rings [33]. New X-ray imaging shows that Kha actually wore six finger rings. In Fig 3, the following rings can be appreciated: i- one rectangular plate-shaped gold ring; ii- one oval plate-shaped gold ring [46]; iii- a gold ring surmounted by a flexible oval scarab; iv. a second oval plate-shaped gold ring [46]; v. a gold ring with a rectangular plate which originally might have been made of faience or stone (only the metal rod holding it is visible as the stone is destroyed) [46]; vi. a gold ring with an oval cartouche [46]. All the described finger rings were popular in the New Kingdom and were worn in both life and in death.

#### Heart scarab (funerary jewellery)

A large heart scarab attached to a long radio-dense rod, possibly a wire of gold or a gold-plated spun chain (Figs 3 and 5), has been identified on Kha’s chest [35]. It can be expected that an inscription with spell 30B from the Book of the Dead is present on the base of the scarab based on parallels. This jewellery was specially made for the burial of Kha.

#### Amulet of Isis (funerary jewellery)

Underneath the collar, is a tyet-amulet (Isis-blood) was observed, normally as described in the Book of the Dead (Spell 156), this amulet was probably made of a red carnelian stone or another red stone (Fig 3). These amulets were a normal part of the funerary jewellery [42,46].

#### Serpent amulet (funerary jewellery)

The ureret-amulet (Egyptian: Wrr.t) in the form of a serpent’s head is generally made of carnelian; here it is unusually placed in the middle of Kha’s forehead (Fig 2). According to tradition, this amulet should be fixed at the neck of the dead to support his breathing in the Afterlife [4,42]. The forehead position in the case of Kha is identical to that of the uraeus serpent in pharaohs’ burials. Fletcher has speculated that by placing the amulet on his forehead, the people of Deir el-Medina honoured their ‘boss’ Kha as a ´local pharaoh´ [55].

#### Golden foil around the upper arms (funerary jewellery)

On each upper arm, Kha displays twisted lengths of thin gold foil (Fig 5). Jewellery made of gold sheet are known from royal burials (e.g. the vulture collar from KV 55 or collars from the burial of Tutankhamun in KV62) [56–58]. Mummies covered with gold foil are a typical of the pre-Amarna, Amarna and Ptolemaic periods. Therefore this type of material in Kha’s burial is consistent with others from the reign of Amenhotep III (i.e. pre-Amarna).

#### Merit’s jewellery

Unlike Kha, Merit only wears the type of jewellery worn in life, with no funerary amulets associated with her body. This might indicate that her death was sudden and unexpected and that her funerary jewellery and coffins were not ready when she passed away. It can be expected that she was dressed in her finest clothes in preparation for the Afterlife. A detailed description of Merit’s jewellery is given below.

#### A broad collar (jewellery worn in life and death)

**Fig 10:**
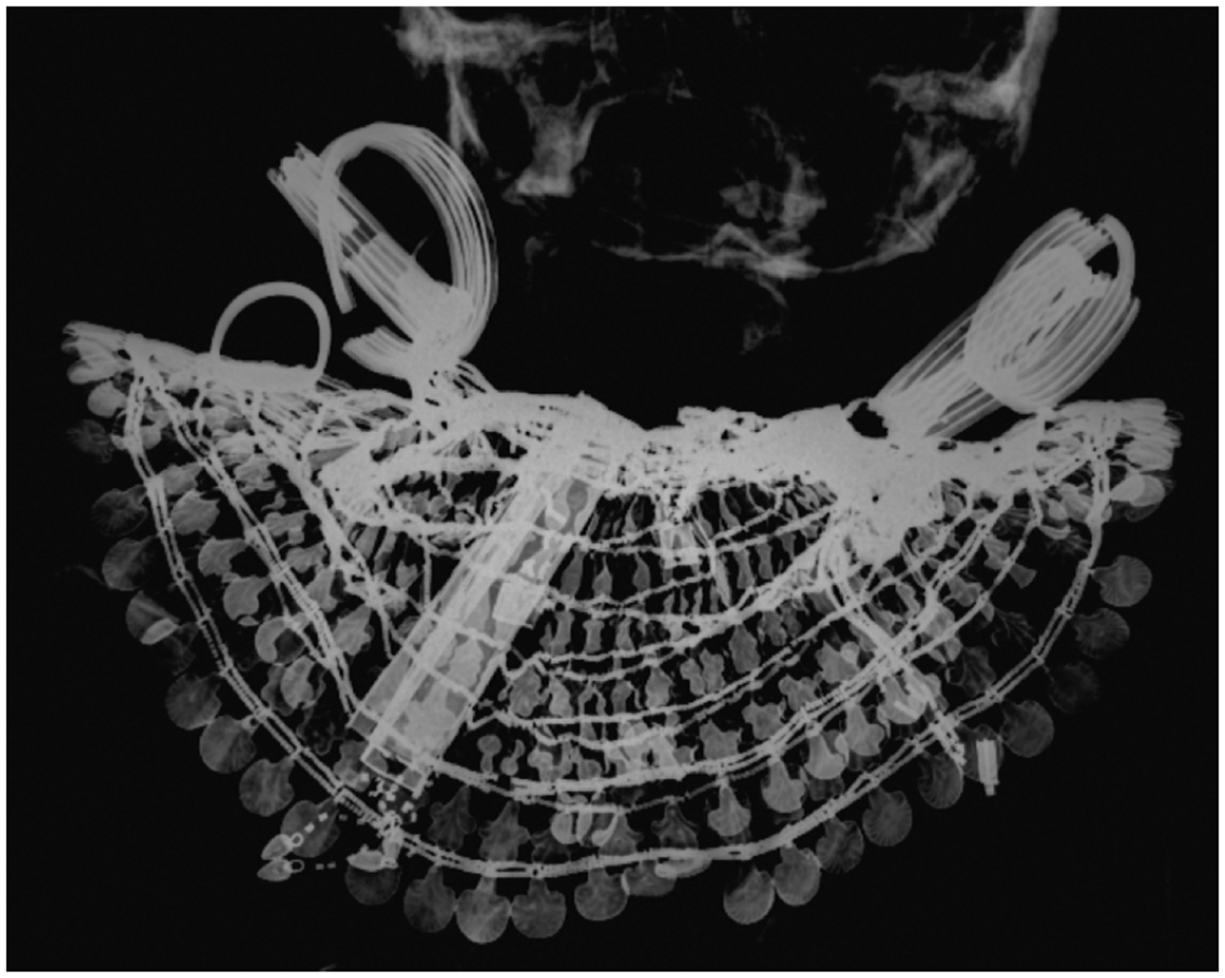
Merit still wears the Usekh-collar

Merit wears a wide collar known as an Usekh-collar, jewellery whose use is testified from the Old Kingdom onwards (Figs 7 and 10) [42,51,59]. The collar is made of gold inter-spaced with gemstones whose exact nature cannot be established (e.g. amethyst, lapis lazuli, carnelian, turquoise faience). A collar with similar elements but a different counterweight was found in the burial of Thutmosis’ III foreign wives [42,46,60–62].

#### Two pairs of golden ribbed-earrings (jewellery worn in life and death)

Merit wears two sets of ribbed-type ear-rings whose typology is known from mid-18^th^ Dynasty (Fig 7) [42,46]. Double-pierced ears were a fashion among elite women of the mid-18^th^ Dynasty, as in the case of the mummies of both Thuyu and the Younger Woman who is most likely Nefertiti [63].

#### Four finger rings (jewellery worn in life and death)

Merit’s hands were adorned with four rings: i. two rings bearing a fixed oval plate; ii. two rings with a flexible oval plate [42,46]. One of the rings with a fixed oval plate fell from her finger and is displaced behind the collar (Fig 7; behind the cranium). This position confirms the hypothesis that Merit’s corpse suffered a post-mortem damage.

#### A girdle (jewellery worn in life and death)

A girdle made of fine beads and metal cowrie shell-shaped parts (Fig 10) is observable level with Merit’s waist. These girdles became popular from the Middle Kingdom onwards and many variations are known, for example the girdle of the 12th Dynasty Princess Sithathoriunet which is made of amethyst beads interspaced with golden leopard heads [42,46], or the girdle worn by one Thutmosis III’s foreign wives- mid-18^th^ Dynasty- which is composed of red glass beads interspaced with gold fish elements [46,60,62].

#### Necklace of fine beads (jewellery worn in life and death)

Merit also wears a three-row necklace made of very fine beads connected by fine golden tubes [33]. The necklace has broken into pieces and parts of its dislocated elements appear near her ankles (Fig 7).

#### A bracelet made of beads (jewellery worn in life and death)

This elegant bracelet follows the same style of the necklace and of the girdle. It is highly likely that they formed a “parure” (Fig 8). The bracelet is made of around 10 rows of fine beads strung between golden elements and a locking end-piece. This type of bracelet was used from the Middle Kingdom onwards, for example a similar bracelet was associated with the burial of Queen Aahotep dating to the beginning of the 18^th^ Dynasty [42].

All the jewellery objects worn by Merit were designed to be worn during her lifetime. From the overall re-appraisal of Merit’s jewellery, excluding the finger rings, it can be hypothesised that her body was adorned with a double set of jewellery combinations: i. the Usekh-collar and one pair of ribbed earrings; ii-the girdle, the bracelet and a second pair of ribbed earrings. However, although New Kingdom’s iconography shows that the large ribbed ear-rings were not normally worn as double earrings, it should again be noted that double-pierced ears, allowing for the wearing of double earrings, were a fashion among elite women of the mid-18^th^ Dynasty, as in the case of both Thuyu and the Younger Woman who is most likely to be Nefertiti [63–67]. Notably, Merit was not equipped with funerary amulets, possibly due to her unexpected death, indicated by the use of her husband’s coffin.

### The outer coffins of Kha and Merit

#### Chemical analysis of the black coatings (gas chromatography-mass spectrometry (GC-MS)) on Kha and Merit’s outer coffins

**Fig 11:**
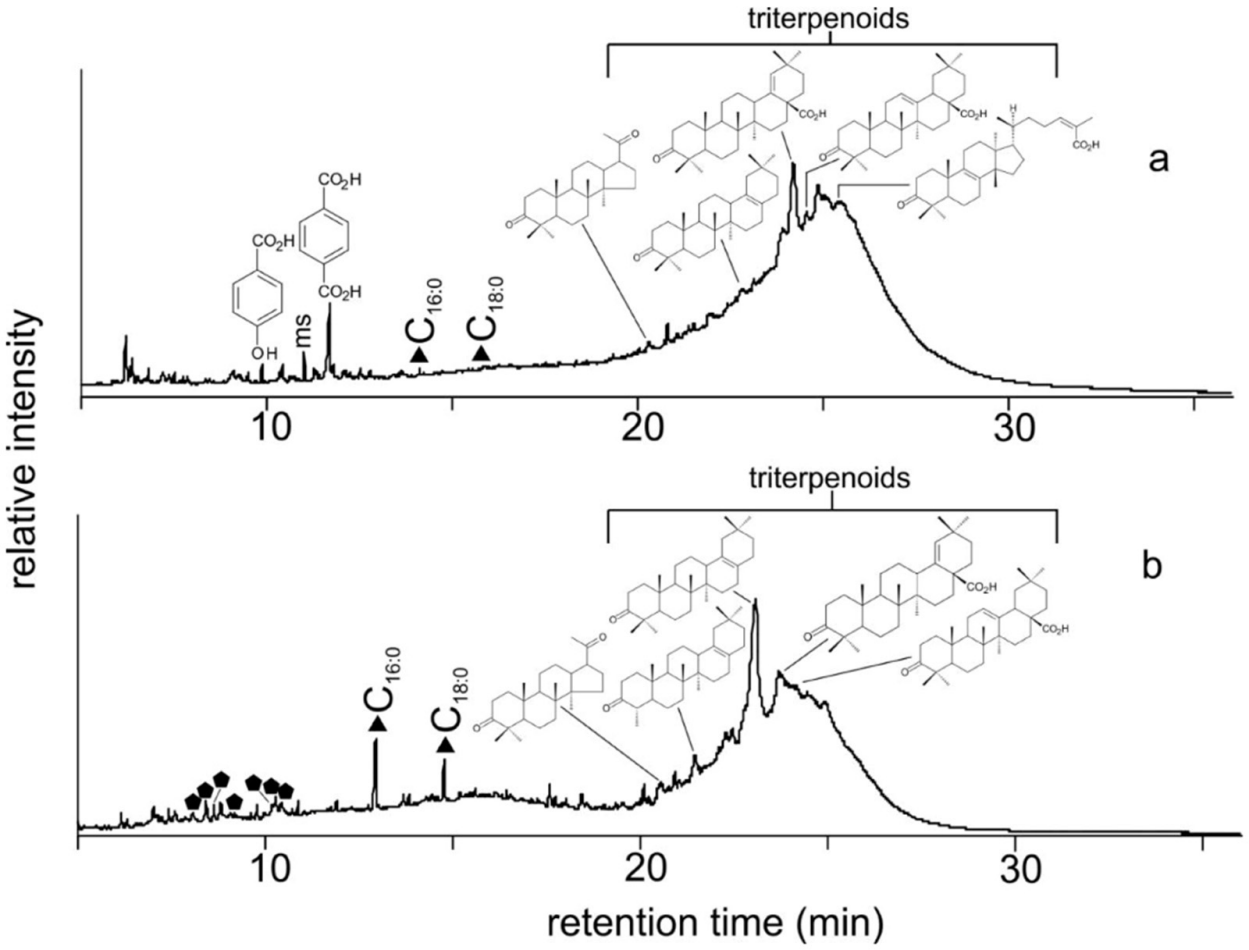
Reconstructed gas chromatography-mass spectrometry (GC-MS) total ion chromatogram (TIC) of the trimethylsilylated total lipid extract of (a) Kha, black coating on his outer coffin and (b) Merit, black coating on her outer coffin. Peak identities (‘n’ indicates carbon chain length; where shown, i indicates degree of unsaturation): filled triangles, C_n:i_ indicates fatty acids; filled pentagons indicate sesquiterpenoids and their derivatives. The letters ms represent a monosaccharide. Also shown are the structures of two aromatic acids identified: 4-hydroxybenzoic acid and terephthalic acid; and the structures of six Pistacia triterpenoid compounds identified: 22,23,24,25,26,27-hexakisnor-dammaran-3,20-dione, 24,28-bisnor-olean-17-en-3-one, 28-nor-olean-17-en-3-one, moronic acid, oleanonic acid and isomasticadienonic acid.

As part of this current study, biochemical investigations (GC-MS) were carried out on two tiny fragments (ca. 0.01 g) taken from Kha’s and Meryt’s outer sarcophagi. The chemical analyses revealed that the shiny black material covering both outer coffins is not bitumen, as previously described [1,2], but a strongly heated “recipe” of mainly Pistacia resin (pitch) with far small amount of balsam and cedar oil/resin (Fig 11a and 11b), possibly mixed with carbon (charcoal), as has been previously suggested as a source of black pigment in the 18^th^ Dynasty [68]. Despite detailed analysis, no evidence for bitumen as a component could be identified.

### Overall observations on the mummification techniques

#### The mummification of Kha

Both the X-rays and previous CT scans reveal that the brain remains in the cranium. Although it is shrunken and has fallen to the back of the cranium, its form is well preserved and immediately recognisable. No damage of the os ethmoidale is visible. CT scans revealed the internal organs within the abdomen and thorax. New X-ray imaging revealed the brain, the bronchus and shrunken lungs (Figs 2 and 5). No inner organs had been removed contrary to the standard practice in royal mummification during the New Kingdom [4,69], explaining why the undisturbed burial had no canopic jars. The preservation of the skeleton is excellent and the eyeballs were visible on the CT-scan as well as the optical nerve and ocular muscles.

#### The mummification of Merit

As in the case of her husband, Merit’s internal organs appear to have been retained within the body including her brain shrunken, but well preserved and recognisable, within the cranium. No damage of the os ethmoidale is visible. Evaluation of the internal organs within the thorax and abdomen was not possible due to severe displacement of the bones of the chest and pelvis. The condition of the skeleton is poor; many bones are broken (probably post-mortem) and displaced. Some form of textile is visible between the legs of Merit, which may represent some form of clothing or may simply be part of the wrappings.

#### Chemical analysis of salts used in the mummification of Kha and Merit (inorganic chemical microanalyses: ‘spot tests’)

White crystalline material from within Merit’s shroud and mummy wrappings was chemically identified as the salt ‘natron’ (sodium carbonate/sodium bicarbonate, sodium chloride and sodium sulphate), regarded as the key ingredient in Egyptian mummification to ‘dry out’ the body.

### Embalming agents used in the mummification of Kha and Merit

#### Chemical analysis of organic residues associated with the linen wrappings from the mummies of Kha wrappings (from base of feet)

**Fig 12:**
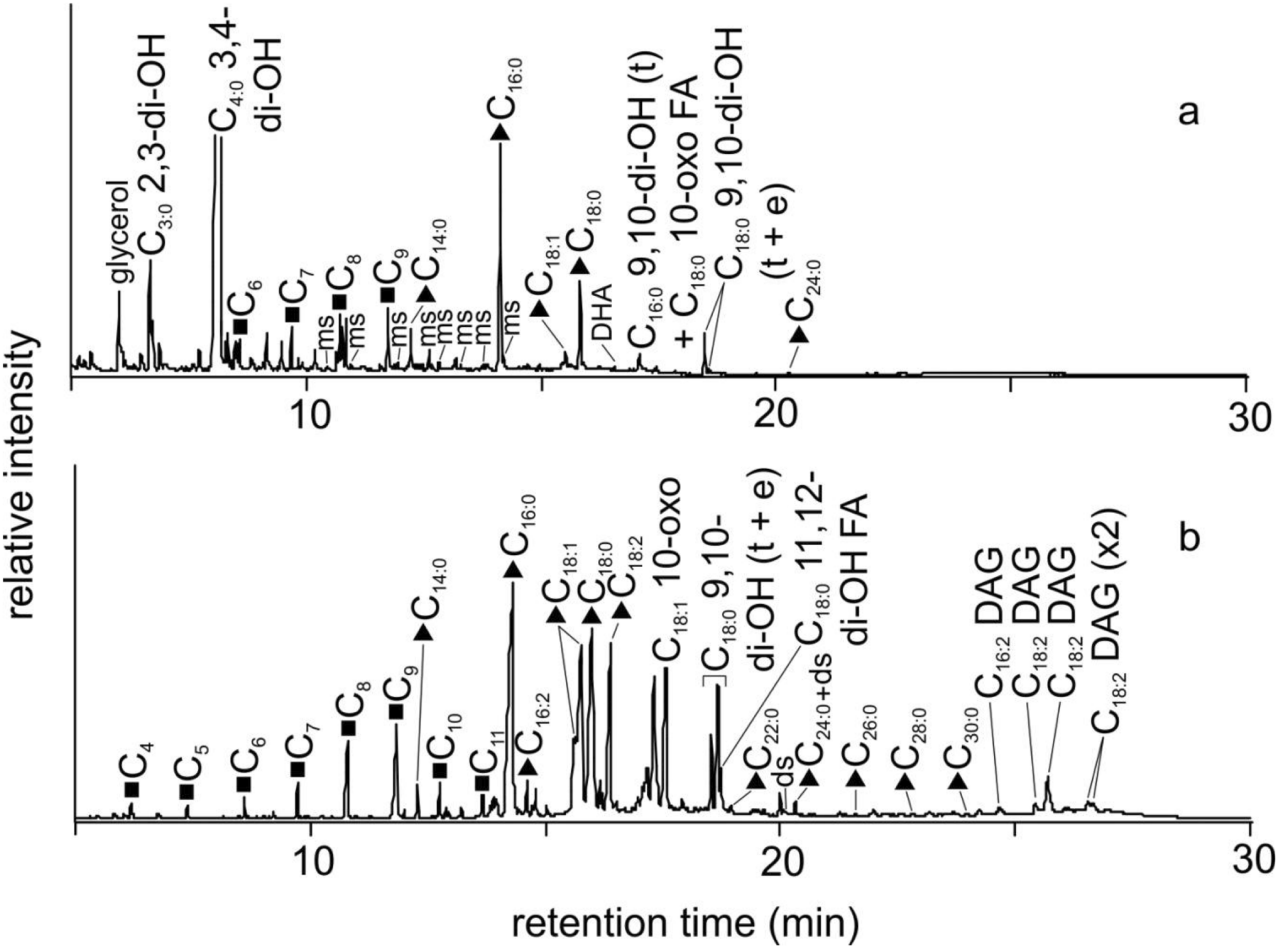
Reconstructed gas chromatography-mass spectrometry (GC-MS) total ion chromatogram (TIC) of the trimethylsilylated total lipid extract of (a) Kha, mummy wrappings (from base of feet) and (b) Merit, mummy wrappings (from base of feet). Peak identities (‘n’ indicates carbon chain length; where shown, i indicates degree of unsaturation): filled triangles, C_n:i_ indicates fatty acids; filled squares, C_n_ indicates α,ω-dicarboxylic acids. C_3:0_ 2,3-di-OH indicates 2,3-dihydroxypropanoic acid (glyceric acid); C_4:0_ 3,4-di-OH indicates 3,4-dihydroxybutanoic acid (2-deoxytetronic acid); C_16:0_ 9,10-di-OH (t) indicates 9,10-dihydroxyhexadecanoic acid (threo isomer); C_18:1_ 10-oxo indicates a 10-oxo-octadecenoic acid; C_18:0_ 10-oxo indicates 10-oxo-octadecanoic acid; C_18:0_ 9,10-di-OH (t+e) indicates 9,10-dihydroxyoctadecanoic acid (threo and erythro isomers); C_18:0_ 11,12-di-OH indicates 11,12-dihydroxyoctadecanoic acid; C_18:2_ DAGs indicate octadecadienoyl diglycerides. The letters ms represent monosaccharides and the letters ds represent disaccharides; DHA represents dehydroabietic acid.

Biochemical analysis (GC/MS) carried out on a linen fragment from Kha’s external wrappings indicates that the textiles were treated with a specific embalming “recipe” consisting of animal fat/plant oil mixed with a small amount of balsam/aromatic plant extract, a plant gum and a conifer resin (Fig 12a).

#### Merit’s mummy wrappings (from base of feet)

Biochemical analysis (GC-MS) of a linen fragment from Merit’s external wrappings revealed an embalming recipe consisting of a highly unusual oil (fish oil) mixed with a small amount of a balsam/aromatic plant extract, a plant gum, a conifer resin and beeswax (bitumen was absent) (Fig 12b).

#### Merit’s red linen shroud

Chemical analysis (GC-MS) of a linen fragment from Merit’s red linen shroud revealed a similar recipe to her mummy wrappings, yet there were also key differences. It consisted of the same highly unusual oil (fish oil), mixed with a small amount of conifer resin and beeswax, but additionally and most notably, Pistacia resin (the ‘balsam’ and gum seen in the wrappings were absent, as, again, was bitumen). The absence of the plant gum and ‘balsam’ on the red shroud perhaps suggests that these were seen as more practical and less symbolic than those impregnating the shroud.

## Discussion

The black coatings on the outer coffins containing the mummies of Kha and Merit had been reported in the literature in both cases as ‘bitumen’ [1,2,70]. However, the findings presented here show that the black ‘paint’ was actually a mixture of ingredients, with bitumen absent. The strong heating observed from the biochemical analyses would have blackened the original resinous mixture producing a ‘pitch’ and giving the coffins their black shiny appearance. The two coatings are notably similar, although Kha has a small amount of plant gum not observed in Merit’s, and Merit has more cedar oil/resin and animal fat/plant oil in her black coating. However, the major component constituting the black shiny coating is a Pistacia pitch, which has previously been identified as the main constituent of black ‘paint’ or ‘varnish’ on other elite funerary object from the New Kingdom [59]. Pistacia and cedar are both regarded as having been expensive commodities coming from the north-eastern Mediterranean. This finding also confirms the strong trading links existing between Egypt and Mitanni (Syria) and the important port of Byblos (Lebanon) at this time.

Observations from the X-ray images’ combined with chemical investigations shed new light on Kha’s and Merit’s mummification procedures and contradict previous statements that neither were embalmed and that essentially no mummification whatsoever took place. On the contrary, not only were both Kha and Merit embalmed with natural products that would have involved significant cost and great effort to obtain (despite Merit’s notably earlier death), but the presence of their preserved, if shrunken, internal organs and moderately well preserved bodies suggests significant efforts were made by their embalmers.

It was, therefore, argued that Kha was embalmed with a shorter and shoddy procedure [29] despite his relative wealth at death. Moreover, it has even been suggested that neither Kha nor Merit were mummified, but merely wrapped in linen [30,31]; this interpretation was also, arguably, ‘corroborated’ by the absence of canopic jars in the burial chamber. Nevertheless, all internal organs: brain, ocular bulbs/ocular nerves, thoracic and abdominal organs- showed a very good state of preservation [29,36]. The non-removal of viscera is not necessarily a sign of low quality mummification; even some known mummies of Queens from the Middle Kingdom were not eviscerated [13,69].

Kha’s “uncommon” thanatological treatment (as well as Merit’s) is not surprising if properly contextualised. Indeed, throughout the 18th Dynasty, irrespective of the economic and status differentials, the vast majority of villagers from Deir el Medina were probably not embalmed in the way normally described. The poorer people buried in the eastern necropolis were simply wrapped in linen without evisceration. Virtually no canopic jars have been found in pre-Ramesside tombs at that site [30].

X-ray images’ observation coupled with chemical investigations shed new light on Kha’s and Merit’s mummification procedures and contradict previous statements that neither were embalmed and no mummification at all took place. On the contrary, not only were both Kha and Merit embalmed with natural products that would have involved significant cost and great effort to obtain (despite Merit’s notably earlier death), but the presence of their preserved, if shrunken, internal organs and moderately well preserved bodies.

Notably, previous reports imply that Merit’s corpse was embalmed “without great care” [30,31], and her brain was said to be fairly well preserved [29] Chemical analysis carried out on a linen fragment from Merit’s external bandages (from the base of her feet) indicates that her linen wrappings were treated with a specific embalming “recipe” which is notably different from Kha’s.

Kha’s external wrappings were treated with an embalming “recipe” consisting of animal fat/plant oil mixed with a small amount of balsam/aromatic plant extract, a plant gum and a conifer resin. The coniferous resin and the “balsam” gave the embalming “recipe” highly preservative—anti-bacterial and anti-insecticidal—properties [20,22,71]

Merit’s embalming “recipe” consists largely of anunusual oil mixed with small amount of ‘balsam’, a conifer resin, beeswax and plant gum, providing at least some anti-bacterial protection. The presence of natron salt within Merit’s outer wrappings confirms Merit’s corpse was treated with what is regarded as the key ingredient of mummification, which again contradicts the presumed poor treatment afforded to her.

## Conclusion

Both mummies were richly decorated with jewellery, and Kha additionally wears funerary amulets. Both probably also wear clothing. They were mummified with no removal of their internal organs (requiring no need of canopic equipment), including the brain, which remained inside the cranium.

The time and effort undoubtedly employed to embalm both Kha and Merit and the use of imported costly resins, notably Pistacia, do not support the view that the two notables were poorly mummified rather they provide the first evidence of an uncommon thanatological treatment applied to a mid-18^th^ Dynasty wealthy couple.

## References

[1] Vassilika E. The Tomb of Kha. Firenze: Scala Group; 2010.

[2] Schiaparelli E. La tomba intatta dell’architeto Cha. Relazione sui lavori della missione archeologica Italiano in Egitto (Anno 1902–1920). Turin; 1927.

[3] Schiaparelli E, Roccati A, Fisher B. La tomba intatta dell’architetto Kha nella metropoli di Tebe-The intact tomb of the architect Kha in the necropolis of Thebes: 2. AdArte; 2008.

[4] Ikram S, Dodson A. The Mummy in Ancient Egypt: Equipping the Dead for Eternity. London: Thames & Hudson; 1998.

[5] Herodotus The Histories. De Silincout A, editor. London: Penguin; 1954.

[6] Diodorus Siculus The Library of History. Oldfather CH, editor. London: Heinemann; 1935.

[7] Strabo The Geography. Jones HL, editor. London: Heinemann; 1932.

[8] Pliny Natural History. Rackham H, editor. London: Heinemann; 1968.

[9] Smith GE. Tutankhamen and the Discovery of his Tomb. London: Routledge; 1923.

[10] Smith GE. Egyptian Mummies. London: Allen & Unwin; 1924.

[11] Cockburn A, Cockburn E, Reyman TA. Mummies, Disease and Ancient Cultures. Cambridge: Cambridge University Press; 1998.

[12] Aufderheide AC. The Scientific Study of Mummies. Cambridge University Press; 2003.

[13] Wade AD, Nelson AJ. Radiological evaluation of the evisceration tradition in ancient Egyptian mummies. Homo. 2013;64: 1–28. 10.1016/j.jchb.2012.11.005 23290862

[14] Sydler C, Öhrström LM, Woitek U, Rühli FJ. CT-based assessment of relative soft tissue alteration in different types of ancient mummies. Anat Rec (special mummy issue). 2015; 10.1002/ar.23144 25998649

[15] Rühli FJ. Magnetic Resonance Imaging of ancient mummies. Anat Rec (special mummy issue). 2015; 10.1002/ar.23150 25998645

[16] Öhrström LM, Fischer B, Bitzer A, Wallauer J, Walther M, Rühli FJ. Terahertz imaging modalities of ancient Egyptian mummified objects and a naturally mummified rat. Anat Rec (special mummy issue). 2015; 10.1002/ar.23143 25998647

[17] Colombini MP, Modugno F, Silvano F, Onor M. Characterization of the balm of an Egyptian mummy from the seventh century B.C. Stud Conserv. 2000;45: 19–19.

[18] Harrell JA, Lewan MD. Sources of mummy bitumen in ancient Egypt and Palestine. Archaeometry. 2002;44: 285–293.

[19] Koller J, Baumer U, Kaup Y, Etspüler H, Weser U. Embalming was used in Old Kingdom. Nature. 1998;391: 343–344. 9450745 10.1038/34809

[20] Buckley S, Evershed RP. Organic chemistry of embalming agents in Pharaonic and Graeco-Roman mummies. Nature. 2001;413: 837–841. 10.1038/35101588 11677605

[21] Buckley SA, Stott AW, Evershed RP. Studies of organic residues from ancient Egyptian mummies using high temperature-gas chromatography-mass spectrometry and sequential thermal desorption-gas chromatography-mass spectrometry and pyrolysis-gas chromatography-mass spectrometry. Analyst. 1999;124: 443–452. 10605875 10.1039/a809022j

[22] Koller J, Baumer U, Kaup Y, Schmid M, Weser U. Analysis of a pharaonic embalming tar. Nature. 2003;425: 784 14574400 10.1038/425784a

[23] Hornung E, Krauss R, Warburton DA, Seidelmeyer SJ, Verner M, Schneider T, et al Ancient Egyptian chronology. Hornung E, Krauss R, Warburton DA, editors. Leiden: Brill; 2006.

[24] Jones J, Higham TFG, Oldfield R, O’Connor TP, Stephen Buckley. Evidence for Prehistoric Origins of Egyptian Mummification in Late Neolithic Burials. PLOS One. 2014;9: e103608 10.1371/journal.pone.0103608 25118605 PMC4132097

[25] Peck WH. Mummies of ancient Egypt In: Cockburn A, Cockburn E, Reyman TA, editors. Mummies, Disease and Ancient Cultures. Cambridge: Cambridge University Press; 1998 pp. 15–37.

[26] Millet NB, Hart GD, Reyman TA, Zimmerman MR, Lewin PK. ROM I: mummification for the common people In: Cockburn A, Cockburn E, Reyman TA, editors. Mummies, Disease and Ancient Cultures. Cambridge: Cambridge University Press; 1998 p. 91105.

[27] Saleem SN, Hawass Z. Variability in Brain Treatment During Mummification of Royal Egyptians Dated to the 18th–20th Dynasties: MDCT Findings Correlated With the Archaeologic Literature. Am J Roentgenol. 2013;200: 336–344. 10.2214/AJR.12.9405 23521476

[28] Saleem SN, Hawass Z. Subcutaneous Packing in Royal Egyptian Mummies Dated From 18th to 20th Dynasties [online]. J Comput Assist Tomogr. 2015; 10.1097/RCT.0000000000000205 25695867

[29] Martina MC, Cesarani F, Boano R, Donadoni Roveri AM, Ferraris A, Grilletto R, et al Kha and Merit: multidetector computed tomography and 3D reconstructions of two mummies from the Egyptian Museum of Turin. Journal of Biological Research Vol LXXX, N 1, Proceedings V World Congress on Mummy Studies. Rubbettino Editore; 2005 pp. 42–44.

[30] Meskell L. Intimate archaeologies: the case of Kha and Merit. World Archaeol. 1998;29: 363–379.

[31] Meskell L. Archaeologies of Life and Death. Am J Archaeol. 1999;103: 181–199.

[32] Davis TM. The Tomb of Ioiya and Touiyou. London: Constable & Co; 1907.

[33] Curto S, Delorenzi E, Spagnotto D. I risultati d’una rilevazione radiografica e grafica su mummie. Oriens Antiq. 1980;19: 147–157.

[34] Delorenzi E, Grilletto R. Le Mummie Del Museo Egizio Di Turino N 13001–13026. Indagine anthropo-radiologica. Milano; 1989.

[35] Curto S, Mancini M. News of Kha and Meryt. JEA. 1968;54: 77–81.

[36] Cesarini F, Martina MC, Boano R, Grilletto R, D’Amicone E, Venturi C, et al Scenes from the Past. Multidetector CT Study of Gallbladder Stones in a Wrapped Egyptian Mummy. RandioGraphics. 2009;29: 1191–1194. 10.1148/rg.294085246 19605665

[37] (FDI). FDI. Two-digit system of designation of teeth. Int Dent J. 1971;21: 104–106.

[38] Trotter M, Gleser GC. Estimation of stature from long bones of American Whites and Negroes. Am J Phys Anthropol. 1952;10: 463–514. 10.1002/ajpa.1330100407 13007782

[39] Pearson K. Mathematical Contributions to the Theory of Evolution. V. On the Reconstruction of the Stature of Prehistoric Races. Philos Trans R Soc London Ser A, Contain Pap a Math or Phys Character. 1899;192: 169–244. 10.1098/rsta.1899.0004

[40] Habicht ME, Henneberg M, Öhrström LM, Staub K, Rühli FJ. Body Height of Mummified Pharaohs Supports Historical Suggestions of Sibling Marriages [online]. Am J Phys Anthropol. 2015; 10.1002/ajpa.22728 25916977

[41] Melcher AH, Holowka S, Pharoah M, Lewin PK. Non-invasive computed tomography and three-dimensional reconstruction of the dentition of a 2,800-year-old Egyptian mummy exhibiting extensive dental disease. Am J Phys Anthropol. 1997;103: 329–40. 10.1002/(SICI)1096-8644(199707)103:3<329::AID-AJPA3>3.0.CO;2-L 9261496

[42] Andrews C. Ancient Egyptian Jewellery. London: The Thrustees of the British Museum; 1990.

[43] Vogelsang-Eastwood GM. Pharaonic Egyptian Clothing. Leiden, New York: E.J. Brill; 1993.

[44] Rutica D, Habicht ME. Die Kleidung der Alten Ägypter. Kleidung, Perücken und Schmuck im pharaonischen Ägypten [in preparation]. 2015.

[45] Watson PJ. Costume of Ancient Egypt. London: B.T. Batsford; 1987.

[46] Müller HW, Thiem E. Die Schätze der Pharaonen. Augsburg: Battenberg; 1998.

[47] Vernier É. Note Sur Les Boucles D’Oreilles Égyptiennes. BIFAO. 1911;8: 15–41.

[48] Binder S. The Gold of Honour in New Kingdom Egypt. Oxford: Aris & Phillips Ltd; 2008.

[49] Stierlin H, Ziegler C. Tanis Vergessene Schätze der Pharaonen. München: Hirmer Verlag; 1987.

[50] Yoyotte J, Ziegler C, Leclant J, Vernus P. Tanis L’Or des Pharaons. Paris: Association Française d’Action Artistique; 1987.

[51] Saleh M, Sourouzian H. Die Hauptwerke im Ägyptischen Museum Kairo. Mainz am Rhein: Philipp von Zabern; 1986.

[52] Desroches-Noblecourt C, Duc M, Eggebrecht E, Hassanein F, Kurz M, Nelson M. Sen-nefer. Die Grabkammer des Bürgermeisters von Theben. Mainz: Philipp Von Zabern; 1986.

[53] Davies N de G. The rock tombs of El Amarna. London: Off. of the Egypt Explor. Fund; 1903.

[54] Martin GT. The hidden tombs of Memphis : new discoveries from the time of Tutankhamun and Ramesses the Great. London: Thames and Hudson; 1991.

[55] Fletcher J. Ägypten—Leben und Sterben im Tal der Könige—(2/2)—Doku/Dokumentation. Deutschland / Frankreich (arte); 2014. Available: https://www.youtube.com/watch?v=DwhzuGZ9Nug

[56] Reeves NC. The Complete Tutankhamun. The King. The Tomb The Royal Treasure. London: Thames and Hudson Ltd; 1992.

[57] Edwards IES. Tutanchamun Das Grab und seine Schätze. GmbH GLV, editor. Bergisch Gladbach; 1978.

[58] James TGH. Tutanchamun. Köln: Karl Müller Verlag; 2000.

[59] Aldred C, Shoucair A. The jewels of the Pharaos: Egyptian jewellery of the dynastic period. London: London : Thames and Hudson; 1971.

[60] Lilyquist C. The Tomb of Tuthmosis III’s Foreign Wives. New York: Metropolitan Museum of Art; 2004.

[61] Habicht ME. Gräber von Pharaonen und Königinnen des alten Ägypten, die noch zu finden sind. Unter dem Siegel der Nekropole. 2014;1.

[62] Winlock HE. The Treasure of Three Egyptian Princesses. New York: Metropolitan Museum of Art; 1948.

[63] Fletcher J. The Search for Nefertiti. New York: William Morrow; 2004.

[64] Gabolde M. L’ADN de la famille royale amarnienne et les source égyptiennes. Egypte Nilotique Méditeranéenne ENiM. 2013;6: 177–203.

[65] Habicht ME. Nofretete und Echnaton: Das Geheimnis der Amarna-Mumien. Leipzig: Koehler + Amelang Gmbh; 2011.

[66] Saleh A. The Royal Mummies. Cairo: Middle East Co; 2010.

[67] Schlögl HA. Nofretete. Die Wahrheit über die schöne Königin. Beck C. H.; 2012.

[68] Lucas A. Ancient Egyptian Materials and Industries. (rev. Harris J.R.), editor. London: Histories and Mysteries of Man; 1989.

[69] Derry DE. Mummification II—methods practiced at different periods. ASAE. 1942;41: 240–265.

[70] Moiso B. Ernesto Schiaparelli e la Tomba di Kha. Turin: AdArte; 2008.

[71] Buckley SA, Clark KA, Evershed RP. Complex organic chemical balms of Pharaonic animal mummies. Nature. 2004;431: 294–9. 10.1038/nature02849 15372029

